# Supplementary material for: Mechanism of the Affinity-Enhancing Effect of Isatin on Human Ferrochelatase and Adrenodoxin Reductase Complex Formation: Implication for Protein Interactome Regulation
Source: Int J Mol Sci. 2020 Oct 14;21(20):7605. doi: 10.3390/ijms21207605 (PMC7593955; doi:10.3390/ijms21207605)
Supplement: Supplementary file 1 [file ijms-21-07605-s001.zip › ijms-956656-supplementary.docx]

Supplementary

Mechanism of the Affinity-Enhancing Effect of Isatin on Human Ferrochelatase and Adrenodoxin Reductase Complex Formation: Implication for Protein Interactome Regulation

Pavel V. Ershov ^1,*^, Alexander V. Veselovsky ^1^, Yuri V. Mezentsev ^1^, Evgeniy O. Yablokov ^1^, Leonid A. Kaluzhskiy ^1^, Anastasiya M. Tumilovich ^2^, Anton A. Kavaleuski ^2^, Andrei A. Gilep ^2^, Taisiya V. Moskovkina ^3^, Alexei E. Medvedev ^1^, Alexis S. Ivanov ^1^

^1^ Institute of Biomedical Chemistry, 10 Building 8, Pogodinskaya Street, 140006 Moscow, Russia; veselov@ibmh.msk.su (A.V.V.); yuri.mezentsev@ibmc.msk.ru (Y.V.M.); evgeniy.yablokov@ibmc.msk.ru (E.O.Y.); leonid.kaluzhskiy@ibmc.msk.ru (L.A.K.); alexei.medvedev@ibmc.msk.ru (A.E.M.); alexei.ivanov@ibmc.msk.ru (A.S.I.)

^2^ Institute of Bioorganic Chemistry NASB, 5 Building 2, V.F. Kuprevich Street, 220141 Minsk, Belarus; tumilovicham@iboch.by (A.M.T.); bio.kovalevs@iboch.by (A.A.K.); agilep@iboch.by (A.A.G.)

^3^ Far East Federal University, FEFU Campus, 10 Ajax Bay, Russky Island, 690922 Vladivostok, Russia; moskovkina.tv@dvfu.ru (T.V.M.)

***** Correspondence: pavel.ershov@ibmc.msk.ru; Tel.: +7-499-246-71-15

*SPR analysis of ADR/Adx complex formation*


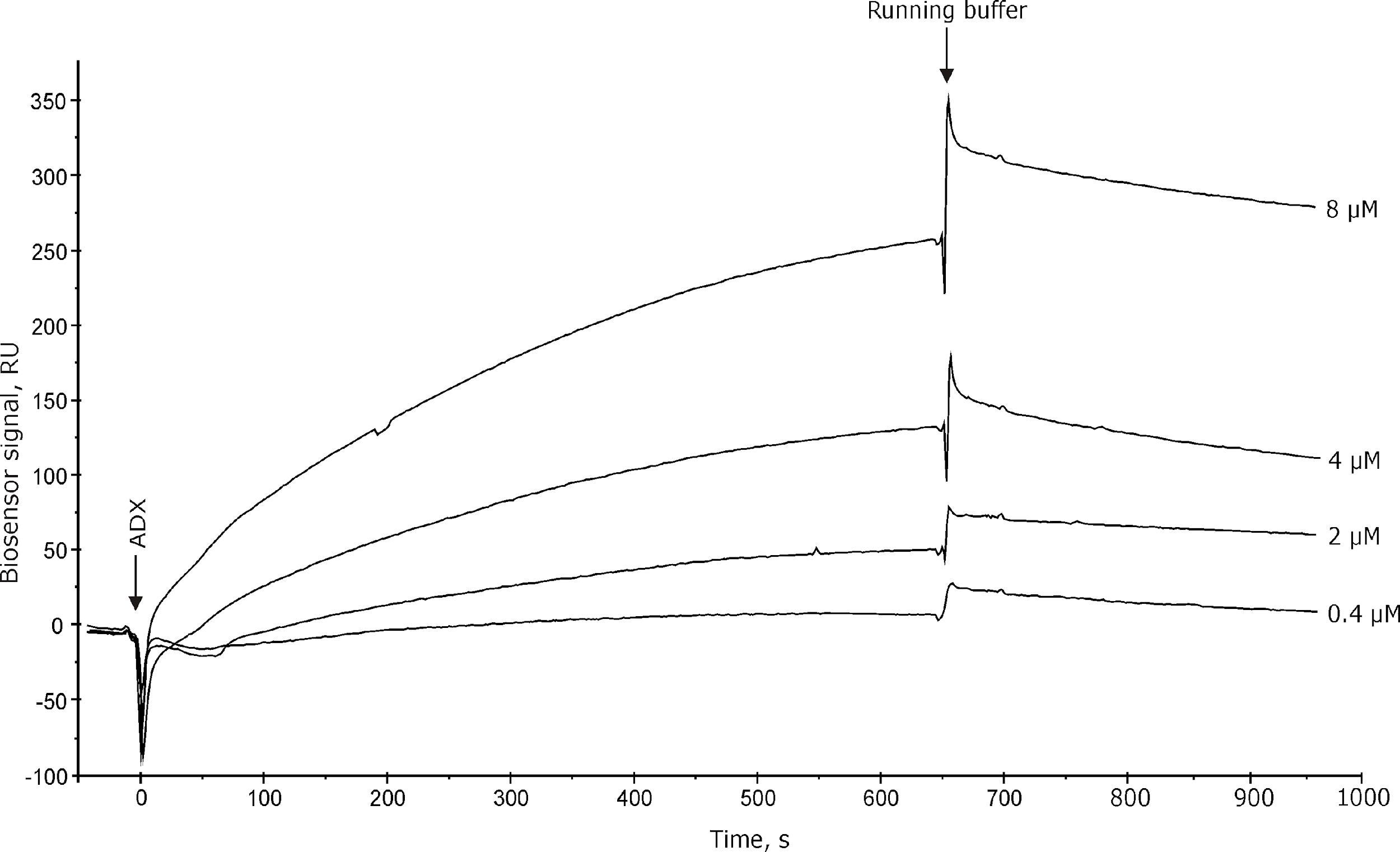


**Figure S1.** SPR sensorgrams of complex formation between adrenodoxin reductase (ADR), covalently immobilized on the optical chip, and adrenodoxin (Adx).

Analyte solutions were injected at a flow rate of 10 µL/min for 10 min. Solution containing 2M NaCl and 0.4% CHAPS was used for the regeneration of the chip surface for 30 s at a flow rate of 50 μL/min. Kd values were calculated in global fitting mode using the BIAevaluation 4.1 software and the Langmuir binding model (1:1 complex formation).

The level of ADR immobilization on the optical chip CM5 was about 4500 RU (corresponds to 4.5 ng/mm2). Stable baseline of biosensor was established by flowing HBS-EP+ running buffer within 2 hours at a flow rate of 10 µL/min. Calculated Kd value of ADR/Adx complex was 3.7 ± 1.1 µМ, which is consistent with previously published Kd value (2.5 µМ) [Lambeth et al., 1984].

*SPR analysis of different proteins binding with 5-aminoisatin, immobilized on the optical chip*


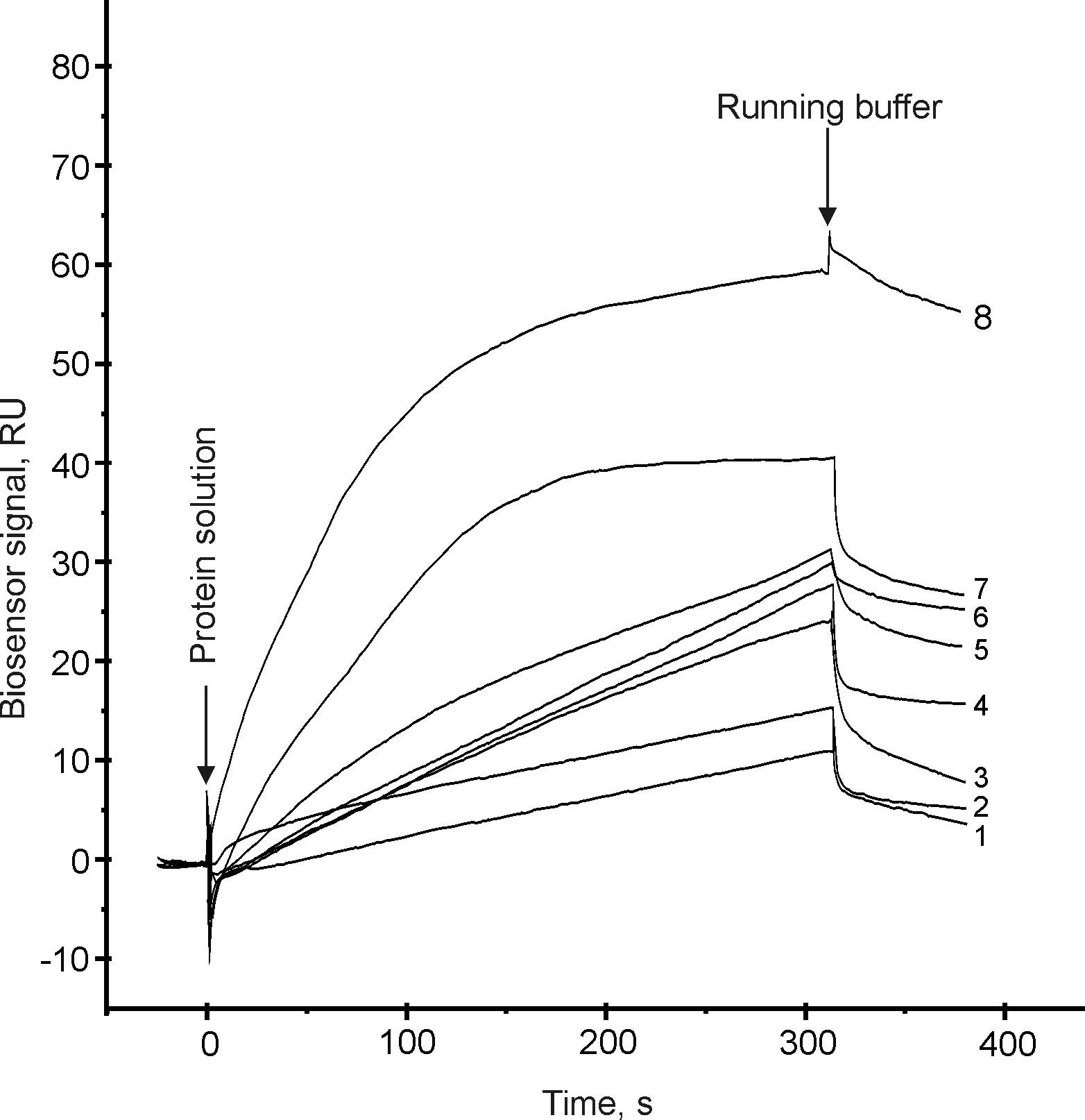


**Figure S2.** Sensorgrams of proteins binding with 5-aminoisatin immobilized on the optical chip. Analysis were performed using Biacore Х-100 biosensor (GE Healthcare, USA) with optical chip CM4. 5-aminoisatin was covalently immobilized in the working flow cell up to 1000 RU according [Buneeva et al., 2010]. Samples of proteins (1 µМ solutions in running buffer) were injected for 5 min at a flow rate of 10 µL/min. For correction of non-specific binding the biosensor signal from the control flow cell (without 5-aminoisatin) was subtracted from the signal from the working flow cell (with the immobilized 5-aminoisatin). Solution containing 2M NaCl and 0.4% CHAPS was used for chip surface regeneration for 30 s at a flow rate of 25 µL/min. The following proteins were tested: BSA (1), beta-1-microglobulin (2), retinol-binding protein 4 (3), beta-2-microglobulin (4), mothers against decapentaplegic homolog 4 SMAD4 (5), alpha-fetoprotein (6), NADPH-dependent adrenodoxin reductase (ADR) (7), ferrochelatase (FECH) (8).

*SPR analysis of isatin influence on FECH/ADR complex formation*


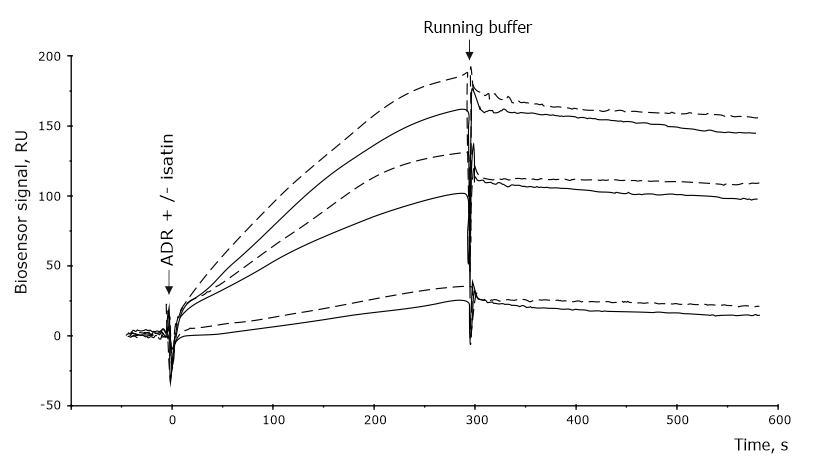


**Figure S3.** Typical SPR sensorgrams of complex formation between immobilized on the chip ferrochelatase (FECH) monomer and three concentrations of NADPH-dependent adrenodoxin reductase (ADR) (bottom-up, 0.5 µM, 5 µM, 20 µM) in the absence (solid line) or presence of 100 µM isatin (dotted line).

*Rebuilding of FECH dimer on the optical chip of biosensor*


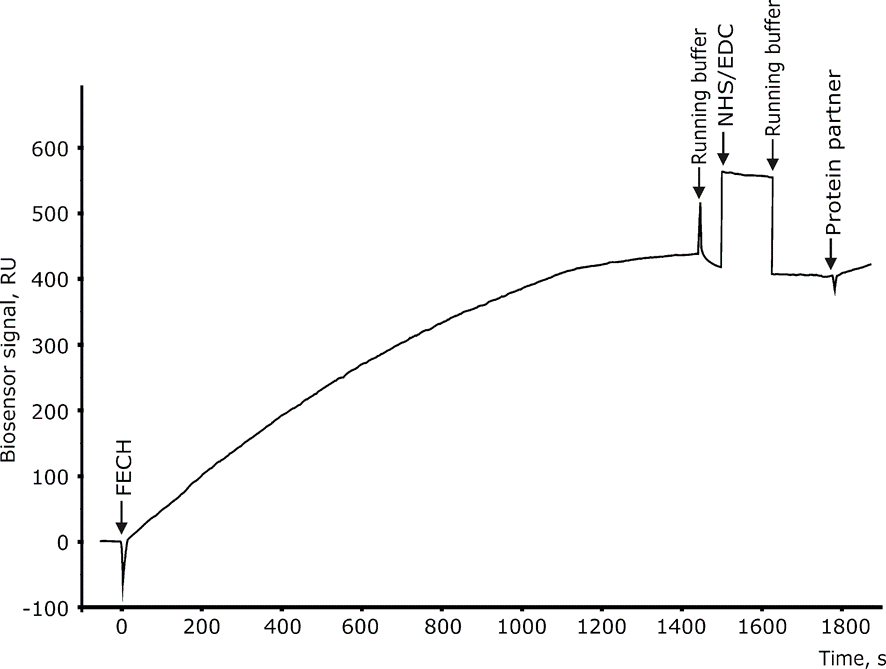


**Figure S4.** The typical SPR sensorgram of the formation of a dimeric form of ferrochelatase (FECH) on the optical chip CМ5 with immobilized FECH monomers. FECH solution in running buffer (5 µM) was injected until an equilibrium biosensor signal was reached.

Right after that, the NHS/EDC mixture (10 μL 400 mM EDC and 10 μL 100 mM NHS) was injected for 2 minutes for chemical stabilization of the FECH dimers by forming additional covalent bonds between FECH subunits in dimers, or between the FECH subunits of adjacent dimers, or between the FECH subunits and dextran matrix.

Literature

Buneeva, O.; Gnedenko, O.; Zgoda, V.; Kopylov, A.; Glover, V.; Ivanov, A.; Medvedev, A.; Archakov, A. Isatin-binding proteins of rat and mouse brain: proteomic identification and optical biosensor validation. *Proteomics* **2010**, *10*, 23–37, doi:10.1002/pmic.200900492.

Lambeth, J.D.; Geren, L.M.; Millett, F. Adrenodoxin interaction with adrenodoxin reductase and cytochrome P-450scc. Cross-linking of protein complexes and effects of adrenodoxin modification by 1-ethyl-3-(3-dimethylaminopropyl)carbodiimide. *J. Biol. Chem.* **1984**, *259*, 10025–10029.

**Table S1.** A fragment of the ZDOCK output file for top 10 FECH/ADR complexes (Header lines).

| **№ of line** | **A** | **B** | **C** | **D** |
| --- | --- | --- | --- | --- |
| 1 | 144 | 1.2 | 1 | - |
| 2 | -2.356194 | 1.756530 | -2.105156 | - |
| 3 | 2.380392 | 2.500101 | -0.120466 | - |
| 4 | lig.job111229.bl.pdb | 3.784 | 12.945 | -18.192 |
| 5 | rec.job111229.bl.pdb | 5.682 | 18.085 | 28.855 |

The columns of line 1: the size N of the NxNxN grid used in the docking (A), the spacing between grid cells (always 1.2 A) (B), and whether the receptor or ligand was fixed during docking (0 = receptor, 1 = ligand) (C). The columns of line 2: the initial rotation of the receptor in Euler angles. The columns of line 3: the initial rotation of the ligand in Euler angles. The columns of line 4: the receptor file (A), followed by its initial translation (x (B), y (C), z (D)) to center. The columns of line 5: the ligand file (A), followed by its initial translation (x (B), y (C), z (D)) to center.

**Table S2.** A fragment of the ZDOCK output file for top 10 FECH/ADR complexes (Prediction lines).

| **№ of complex** | **1** | **2** | **3** | **4** | **5** | **6** | **ZDOCK score** |
| --- | --- | --- | --- | --- | --- | --- | --- |
| I | -2.617994 | 2.404280 | -0.174044 | 11 | 38 | 9 | 1151.120 |
| II | 0.523599 | 1.756530 | -2.105156 | 31 | 22 | 142 | 1053.086 |
| III | 1.047198 | 0.929350 | -2.910676 | 32 | 1 | 6 | 1043.929 |
| IV | 1.570796 | 1.128190 | -2.288622 | 13 | 40 | 3 | 1035.133 |
| V | -3.141593 | 2.863997 | 0.549740 | 31 | 24 | 140 | 1032.254 |
| VI | -2.879793 | 2.108018 | 1.311430 | 28 | 13 | 120 | 1029.320 |
| VII | 2.356194 | 0.618092 | 2.539118 | 18 | 36 | 123 | 1019.748 |
| VIII | 2.617994 | 0.591903 | 2.010408 | 28 | 29 | 135 | 998.296 |
| IX | -2.879793 | 2.251330 | -2.330739 | 118 | 1 | 2 | 997.355 |
| X | 0.785398 | 2.251330 | -2.330739 | 27 | 30 | 135 | 989.781 |

Columns 1-3 are the Euler angles (in radians) for rotating the ligand, columns 4-6 are the grid positions describing the translation of the ligand with respect to its starting point.
